# Editorial Comment: Self-perception, quality of life and ease of catheterization in patients with continent urinary diversion with the mitrofanoff principle

**DOI:** 10.1590/S1677-5538.IBJU.2019.0388.1

**Published:** 2020-07-31

**Authors:** Antonio Carlos Moreira Amarante

**Affiliations:** 1 Hospital Pequeno Príncipe Departamento de Cirurgia Pediátrica Curitiba PR Brasil Departamento de Cirurgia Pediátrica, Hospital Pequeno Príncipe, Curitiba, PR, Brasil

## COMMENT

The Mitrofanoff principle came to meet the needs of patients and urologists at the seventies decade. It made easier the intermitent self catheterization by the neurological impaired patients stablishing a way to empty the bladder without the need of a huge mobilization specially for the wheelchair bound patients or their caregivers.

As the numbers of proceedings raised, the numbers of complications went up too. This is well related in most of the papers published about the Mitrofanoff technique.

The high numbers of complications made us think about the satisfaction or dissatisfaction with the procedure and what is the quality of life of these patients . Very little has been done in this sense. I found only one paper at PUbMed with the analysis of the quality of life of these patients (1). This has a similar number of subjects but it only uses the SF36^®^ health survey V2 that measures 8 health concepts compared against published data for the normal population. Their results are similar to the paper presented by the authors.

The paper describes the satisfaction of the patients in respect to the stoma, in spite of the relatively high numbers of small complications that may have needed surgical treatment or medical attention.

In the present paper the sample is small (22 patients), the ages have a big interval variation ( 5 to 76 years), the follow up varies from 8 to 84 months and the basic diagnosis of the patients varies between neurogenic bladder, urethral strictures,extrophy, cloacal anomalies and other diagnossis (2). This may be a draw back on the analysis of the answers because the patients may be in diferent phases of life, may have diferent sensibility and may have different body image making the sample not very homogeneous.

Anyway, the most importante information is that 95% of the patients would recommend the procedure to a friend or to somebody with the same problem.

This may need more studies with more numbers and a more homogeneous sample to have definitive answers. This paper may be a kick off on this direction.
